# Surface-Enhanced Raman Spectroscopy on Gold Nanoparticle for Sperm Quality Discrimination

**DOI:** 10.3390/molecules30091876

**Published:** 2025-04-23

**Authors:** Yeira P. Lopez-Lora, Nataly J. Galán-Freyle, Natally Vidal-Figueroa, Antony A. Cardozo-Puello, Antonio J. Acosta-Hoyos, Guido Parra-Anaya, Elvin S. Lebrón-Ramírez, Fabián Espitia-Almeida, Samuel P. Hernández-Rivera, Maximiliano Méndez-López, Ornella Fiorillo-Moreno, Karin Rondon-Payare, Leonardo C. Pacheco-Londoño

**Affiliations:** 1 Facultad de Ciencias Básicas y Biomédicas, Life Science Research Center, Universidad Simón Bolívar, Barranquilla 080002, Colombia; yeira_7@hotmail.com (Y.P.L.-L.); nataly.galan@unisimon.edu.co (N.J.G.-F.); natally.vidal@upr.edu (N.V.-F.); antony.cardozo@upr.edu (A.A.C.-P.); antonio.acosta@unisimon.edu.co (A.J.A.-H.); fabian.espitia@unisimon.edu.co (F.E.-A.); ext.ornella.fiorillo@colsanitas.com (O.F.-M.); krondon@unimagdalena.edu.co (K.R.-P.); 2 Instituto de Reproducción Humana Procrear, Barranquilla 080020, Colombia; gparra@procrear.com.co; 3 ALERT DHS Center of Excellence for Explosives Research, Department of Chemistry, University of Puerto Rico-Mayagüez Campus, Mayagüez, PR 00681, USA; elvin.lebron1@upr.edu (E.S.L.-R.); samuel.hernandez3@upr.edu (S.P.H.-R.); 4 Grupo de Química y Biología, Departamento de Química y Biología, Universidad del Norte, Km 5 Vía Puerto Colombia, Barranquilla 080001, Colombia; maximilianom@uninorte.edu.co; 5 Clínica Iberoamérica y Clínica El Carmen, Barranquilla 080020, Colombia; 6 Grupo de Salud Familiar, Facultad de Ciencias de la Salud, Programa de Medicina, Universidad del Magdalena, Santa Marta 470004, Colombia

**Keywords:** sperm-quality, Raman, gold-nanoparticles, male-factor, PLS-DA, SERS

## Abstract

Spermatozoa were isolated from the seminal fluid using washing (wash), post-capacitation (POS), and swim-up (SU) techniques, followed by analysis through Surface-Enhanced Raman Spectroscopy (SERS). Density gradient and swim-up methods were applied to 35 semen samples to validate sperm quality. The resulting spectra showed notable variations at 408 cm^−1^ (S–S stretch attributed to lysozyme) and 728 cm^−1^ (associated with DNA alterations and methylation). These spectral markers were incorporated into partial least squares discriminant analysis (PLS-DA) models to distinguish among sperm populations prepared by different methods. One PLS-DA model differentiated wash from POS and SU, attaining 86% sensitivity and 91% accuracy. Another model distinguished between POS and SU, achieving 77% sensitivity and 74% accuracy. The combined use of SERS and multivariate analysis offers a promising alternative for assessing sperm quality, supported by motility assessments in 35 validated samples. This approach could enhance both the accuracy and efficiency of reproductive diagnostics.

## 1. Introduction

Infertility and impaired fecundity have been concerns throughout history and remain a significant clinical challenge, affecting 8–12% of couples worldwide. Approximately 40–50% of these infertility cases are attributed to male-factor infertility, and around 2% of men exhibit suboptimal sperm parameters [1]. Semen analysis—commonly referred to as a spermogram—plays a pivotal role in population-based research on male reproductive health by assessing the functional status of the testes and accessory sex glands. In clinical practice, semen analysis is a standard initial test for evaluating a patient’s fertility potential [2]. However, classifying men based on standard semen parameters has inherent limitations. The traditional analysis process consists of sample collection, macroscopic evaluation, and microscopic examination of key sperm characteristics, including volume. Estimating semen volume is essential for calculating the total sperm count (sperm concentration multiplied by volume), which differs from sperm concentration alone [3].

Sperm motility is another crucial parameter, as it indicates fertilization capability. Motility is typically graded from fast and progressive (grade a) to slow but progressive (grade b), non-progressive (grade c), or immotile (grade d) [4]. Additionally, assessing sperm vitality differentiates between live and dead immotile sperm [5]. The morphology of the head, mid-piece, and tail is also vital for predicting fertilization potential and the likelihood of spontaneous conception, serving as a primary indicator of a patient’s reproductive status [6]. The World Health Organization’s guidelines, presented in the “WHO laboratory manual for examining and processing human semen”, are widely recognized as the gold standard for semen analysis. Nonetheless, the operator-dependent nature of these evaluations can introduce errors. Recent research exploring automated analysis suggests a pathway to more precise and accurate sperm measurements [7].

When Raman Spectroscopy (RS) is combined with metallic nanoparticles (usually gold or silver), the phenomenon known as Surface-Enhanced Raman Spectroscopy (SERS) arises. This effect occurs because metallic nanoparticles generate enhanced electromagnetic fields (due to the excitation of surface plasmons), which significantly amplify the Raman signal. Thanks to this amplification, the sensitivity of the Raman technique is greatly increased, enabling the detection and detailed analysis of metabolites and biomolecules that would otherwise be difficult to observe using conventional Raman spectroscopy. Consequently, SERS provides a powerful tool for investigating biological processes and developing applications [8,9,10] in fields such as biology [11,12], medicine, pharmacology, and biotechnology [13].

RS has emerged as an innovative and powerful tool for analyzing the biochemical properties of sperm, providing insight beyond the scope of conventional semen analysis. It furnishes detailed information on the chemical composition and molecular structure of tissues [14,15]. Researchers have successfully employed RS to distinguish between normal and abnormal semen samples by identifying specific spectral signatures in sperm [7]. As a laser scattering technique, RS provides a chemical “fingerprint” of a sample’s molecular constituents, and recent advances in confocal microscopy further enhance its ability to detect and localize molecular changes [16,17]. Nanoparticles, with their unique properties, are increasingly utilized in various fields, and their incorporation in RS holds promise for addressing male infertility [7,18].

Despite extensive analysis methods, unexplained male infertility (UMI) continues to challenge the clinical community, affecting men whose semen parameters meet World Health Organization standards [19]. While morphology and motility are central to selecting sperm in assisted reproductive technology (ART), these parameters alone cannot fully determine fertilization potential [20]. Capacitation is a pivotal step in fertilization, and sperm function tests gauge the responsiveness of spermatozoa to this process. Consequently, sperm selection is critical in ART procedures to maximize pregnancy outcomes [21]. Under natural conditions, only the best gametes undergo a stringent selection process en route to the oocyte.

Sperm preparation in ART commonly employs techniques such as swim-up (SU) and Density Gradient Centrifugation (DGC). Research indicates that DGC produces higher-quality sperm for intrauterine insemination (IUI) and in vitro fertilization (IVF) [22,23,24], whereas SU effectively isolates motile spermatozoa [25]. These methods can be employed separately or jointly to enrich the sperm population. In this study, we propose an alternative, more rapid, and highly sensitive approach—SERS—applied to three sperm populations obtained via density gradient. Using gold nanoparticles and a partial least squares discriminant analysis (PLS-DA) model, we aimed to discriminate between normal and abnormal spermatozoa in a cohort of patients undergoing assisted reproductive technology.

## 2. Results

### 2.1. Spermogram Analysis: Conventional Semen Parameters

Semen parameters for all studied samples (*n* = 35) were evaluated by determining the sperm concentration per ml, total concentration, motile spermatazoa (motility categories (A, B, C, D), morphology, and semen volume. Table 1 shows some descriptive statistics parameters of all 10 studied semen parameters for all patients.

### 2.2. Characterization and Surface Raman Enhancement Spectroscopy Activity

**Figure 1:**
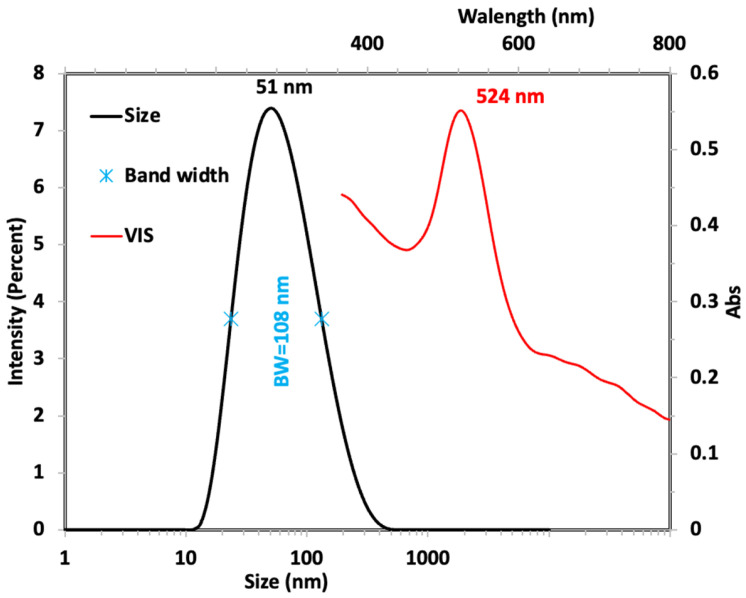
Size distribution graph and absorption spectrum for AuNPs.

AuNPs have an excess of citrate due to synthesis; to clean the excess, these were centrifugated to 6000 rpm, the supernatant was discarded, and the AuNPs were resuspended in the same volume. The AuNPs size was measured by Dynamic light scattering (DLS), optical properties (plasmon resonance) by UV-visible spectroscopy, and SERS activity by Raman spectroscopy was verified by 2-Mercaptoethanol as a target molecule. The AuNPs size was 27 ± 11 nm, and the plasmon resonance was observed at 524 nm, as shown in Figure 1. AuNPs were synthesized following the protocol [26] of some modifications.

### 2.3. PLS-DA Classification Model

PLS-DA models were created with SERS spectral preprocessed by standard vector normalization (SNV). SNV normalization is a widely used data preprocessing technique that corrects for additive and multiplicative variations in spectral measurements. In practice, each spectrum’s mean intensity is subtracted from every point, and the result is then divided by that spectrum’s standard deviation. This transformation centers each spectrum at zero mean with a unit standard deviation, effectively removing baseline offsets and normalizing intensity differences. By mitigating these variability sources—such as differences in sample thickness or laser power—SNV ensures that subsequent comparisons or analyses focus on the intrinsic spectral features rather than extraneous artifacts.

**Figure 2:**
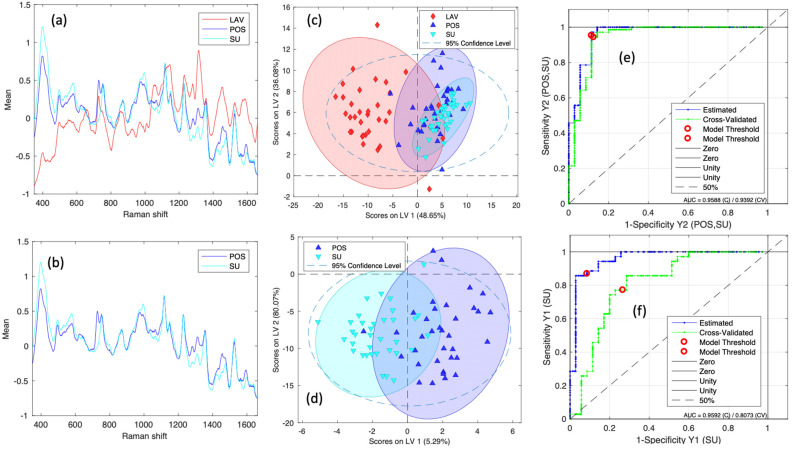
(**a**) Average spectra for the classes LAV, POS and SU. (**b**) Average spectra for the classes POS and SU. (**c**) scatter plot for the score of latent variables 1 and 2 of the first model. (**d**) scatter plot for the score of latent variables 1 and 2 of the second. (**e**) ROC curve for the firs model. (**f**) ROC curve for the second model.

Figure 2a,b shows the SERS spectrum average for wash sperm (red line), sperm post-capacitation (dark blue line), and swim-up sperm (light line). This figure shows the spectral difference between the three different sperm classes; a difference in relative intensity is observed in the position of the bands. Two PLS-DA models were generated, and the confusion matrix and performance parameters for binary models are shown in Table 2. The first model predicts two groups, POS, SU vs. Wash (model 1), and the second model predicts between POS and SU (model 2), which were evaluated using the parameters of a confusion matrix, such as sensitivity (SEN) and specificity (SPE) of the calibration, validation, and prediction model. The results were sensitivity of cross-validation for the first model group—POS, SU vs. wash (SenCV) 0.94 (94%) sensitivity, prediction (SenPred) 0.86 (86%), and accuracy 0.91 (91%)—and the second model—(POS, SU) (SenCV) 0.71 (71%) sensitivity, prediction (SenPred) 0.77 (77%), and accuracy 0.74 (74%). The models show good discrimination, demonstrating that sperm separation by density gradient generates different sperm with differences in their biomolecular composition. Figure 2c,d show only the first two latent variables (LVs) for visualization, even though the first model uses four LVs and the second uses six. Because classification relies on all LVs, this 2D plot is merely a projection that omits higher-dimensional information. As a result, some data points may appear to overlap in 2D space, yet they can still be clearly classified when considering all LVs. These first two LVs contribute the most to the model and, therefore, illustrate a clear separation.

Another parameter measured was the probability of sensing AUC from the curve (see Figure 2e,f) for the two models; the AUC was 0.93 and 0.81 for the cross-validation for the first and second models, respectively. A receiver operating characteristic curve (ROC) is a graphical plot that illustrates the diagnostic ability of a binary classifier system as its discrimination threshold is varied. AUC measures the model’s ability to classify the outcomes correctly. We see that 81% of sperm show differences between SU and POS.

**Figure 3:**
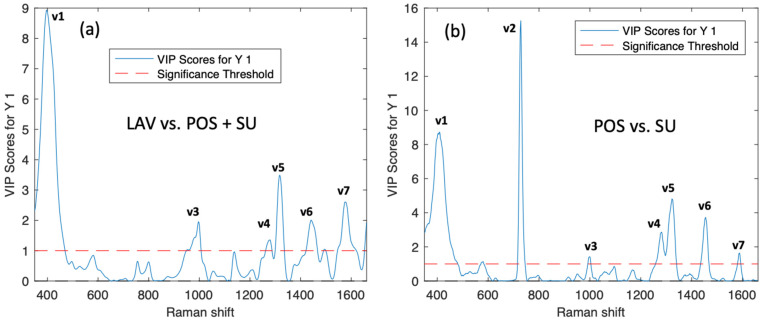
(**a**) Average spectra for the classes and the VIP score for the first model and (**b**) second model.

The variable importance in projection (VIP) score was calculated to find which SERS bands are the most significant in-class differentiation. This was calculated by means of the weighted sum of the squared correlations between the components and the original variable, whose weightings correspond to the percentage variation explained by the PLS-DA component in the model [27]; the VIP scores help us to identify the most important spectral regions that contribute to the optimal discrimination performance of the model [28]. In the VIP scores of the Raman spectra matrix of sperm samples for the classification model, seven bands were identified, contributing to the classification model with more weight than others (see Figure 3).

The SERS sperm spectrum evidences the biochemical changes induced by separating the different sperm for the density gradient and swim-up technique. These differences are observed principally in the bands associated with nucleic acids and protein/lipid content [14]. The peak assignment for the Raman bands with high significance are reported in Table 3.

The total Raman Spectra of seminal plasma showed significant peaks at 408, 728, 1000, 1280, 1319, 1455, and 1575 cm^−1^. As discerned from the analysis, the paramount spectral signal identified at 728 cm^−1^ is predominantly linked to DNA characteristics. In literature reports, this particular signal is often ascribed to deoxyadenosine [29]. Notably, two distinct peaks ranging between 420 and 490 cm^−1^ for the post-capacitation (POS) samples are observed within this spectral region, as depicted in Figure 2b. This bifurcation, however, simplifies into a singular peak at 728 cm^−1^ in the swim-up (SU) samples. The spectral region in question indicates modifications within the DNA structure, including alterations resulting from the methylation process [30]. Based on this, we can attribute the differentiation to damage present in the DNA. The spectral signature at 408 cm^−1^ is attributed to the S–S stretching vibration found in Lysozyme [31], while the peak at 1000 cm^−1^ is associated with the symmetric stretch of the DNA phosphate backbone. This particular peak indicates biochemical changes within DNA [14] and has been previously recognized as a marker for localized DNA damage [29]. The spectral region spanning 1200–1600 cm^−1^ is typically linked to the molecular vibrations of proteins and lipids, specifically the CH and CH_2_ groups [21]. However, the distinct peak at 1280 cm^−1^ corresponds explicitly to the asymmetric stretching of the PO_4_^3−^ group within the DNA phosphate backbone [32]. Additionally, the band at 1319 cm^−1^ is designated for the vibrational modes of guanine, along with CH_3_ and CH_2_ wagging movements found in nucleic acids [33]. The peak appearing at 1455 cm^−1^ is identified with the bending modes of CH_2_ and CH_3_ in tryptophan [7], and the band observed at 1575 cm^−1^ originates from the phosphate stretching vibrations characteristic of DNA and RNA [21].

## 3. Discussion

The different methods of separation, spermatic density gradient centrifugation (DGC) and swim-up are among the most used sperm selection techniques in clinical practice in the assisted reproduction techniques laboratory vary in their ability to separate spermatazoa, possessing nuclear anomalies from those with normal [22,34]. The male reproductive cells, spermatozoa, are pivotal in fertilization, and their composition plays a crucial role in reproductive health. In recent years, applying analytical, biochemical, and spectral analysis techniques has revolutionized our understanding of spermatozoa composition [19]. This approach allows us to identify changes in the biochemical, establishing distinct cell component signatures for healthy control and among groups of different sperm populations.

It was demonstrated that the combination of Surface-Enhanced Raman Spectroscopy (SERS) and multivariate analysis is an effective method of distinguishing between sperm populations prepared using different methods. The presence of specific spectral markers, such as the bands at 408 cm^−1^ and 728 cm^−1^, was instrumental in differentiating sperm quality beyond conventional parameters, such as motility, thereby providing molecular-level insights into sperm biochemistry.

A comparison of our findings with those of previous studies reveals Wiwanitkit et al. [18] as the first to explore the toxicological effects of gold nanoparticles on spermatozoa. Their results showed nanoparticle penetration into the sperm head and tail, leading to reduced motility and fragmentation. While the focus of the aforementioned study was on toxicity, the present study utilises gold nanoparticles as enhancing agents in SERS, with no evidence of acute toxicity, thereby emphasising their potential for analytical purposes under controlled conditions.

In a similar manner, Sánchez et al. [29] utilized conventional Raman microspectroscopy to detect oxidative DNA damage in sperm. The intensity ratio between the 1050 and 1095 cm^−1^ peaks was utilized as a marker of DNA fragmentation, which was then correlated with the results obtained from flow cytometry. Despite the absence of SERS in their methodology, the outcomes of their study corroborate the notion that Raman spectroscopy serves as a non-destructive instrument for the assessment of molecular damage in sperm, a conclusion that is in alignment with the identification of DNA-associated changes at 728 cm^−1^ as reported by our group. The study by Liu et al. [15] applied Raman spectroscopy to testicular tissue to distinguish between complete and incomplete spermatogenesis in seminiferous tubules. While the study’s context was ex vivo tissue evaluation, its findings underscore the sensitivity of Raman to subtle biochemical changes, akin to our capacity to differentiate sperm populations based on preparation methods. In a subsequent study, Liu et al. [17] employed real-time Raman microspectroscopy to distinguish between live sperm bound to the zona pellucida (ZP) and unbound sperm. Spectral differences were reported in regions such as the acrosome and nucleus, which are linked to sperm functionality. This approach adopted by Liu, as well as the present study, underscores the potential of Raman-based techniques to discern functional characteristics in sperm cells.

In conclusion, our study has shown that SERS is an invaluable analytical technique for biological and medical fertility research, particularly in assessing sperm quality. It facilitates the identification of both biochemical and morphological alterations in specimens, negating the need for labeling or extensive preparation. The efficacy of SERS in clinical andrology and research will ultimately depend on its ability to precisely pinpoint spermatozoa exhibiting superior physiological traits while ensuring the methods remain noninvasive and nondestructive to the viability of live sperm cells.

## 4. Materials and Methods

### 4.1. Semen Collection and Semen Analysis

The study received ethical approval from the Instituto de Reproducción Humana Procrear Ethics Committee and the Universidad Simón Bolívar Ethics Committee in Barranquilla, Colombia. Clinical specimens were obtained from 35 patients who had observed 2 to 5 days (48–120 h) of sexual abstinence. The collection was performed via masturbation using a sterile, semen-safe container. Samples were then allowed to liquefy completely at 37 °C for 30 min before further analysis. Following liquefaction, semen parameters were assessed.

This study focused on evaluating the physical characteristics of sperm, including volume and viscosity. Semen volume was measured using a graduated pipette. Sperm concentration and motility were determined using a 5 μL sample of liquefied semen, which was analyzed in a Makler chamber (Sefi-Medical, Haifa, Israel) and examined with an Eclipse Ci-L plus microscope (Nikon, Tokyo, Japan). Sperm motility was categorized as ‘progressively motile’, ‘non-progressively motile’, or ‘immotile’. A minimum of 100 intact spermatozoa were counted across five fields to assess motility.

For morphology evaluation, a smear was prepared from a well-mixed sperm suspension (5–15 μL) and placed onto a microscope slide. After air-drying, the slides were fixed and stained using the Diff-Quik staining method. Two hundred spermatozoa per replicate were counted under bright field illumination at ×1000 magnification with oil immersion. The percentage of normal morphology was calculated based on strict criteria, with a normality threshold of 4% as per World Health Organization (WHO) standards [3]

### 4.2. Separation of Spermatozoa, Density Gradient Centrifugation, and Swim-Up

Twenty μL of semen were taken from the oh whole fresh semen, diluted and washed with Puresperm^TM^ medium (Nidacon, Mölndal, Sweden), then centrifuged (Thermo Scientific Centrifuge, New York, NY, USA) at 1600 (revolutions per minute) × 5 min. After removing the supernatant, the pellet was suspended in 0.5 mL of Sperm Rinse medium (Vitrolife, Gothenburg, Sweden) and evaluated to classify the first sample wash fresh sperm group (wash). The DGC procedure was performed using one mL each of density Gradient Centrifugation 90, 45% Sperm Grad^TM^ medium (Vitrolife, Gothenburg, Sweden), and 2 mL of whole semen in three layers was prepared in a 12 mL Nunc conical tube (Thermo Scientific Nunc, New York, NY, USA), then centrifuged (Thermo Scientific Centrifuge, New York, NY, USA) at 1600 (revolutions per minute) × 15 min. After the supernatant was removed, the pellet was suspended in 3 mL of Sperm Rinse medium (Vitrolife, Gothenburg, Sweden) and centrifuged (Thermo Scientific Centrifuge, New York, NY, USA) again at 1100 g for 10 min; after the supernatant was removed, 20 μL of wash sperm was taken from the sperm post-capacitation, diluted and washed with 0.5 mL of Puresperm^TM^ medium (Nidacon, Mölndal, Sweden), and tested to classify the second group sample post-capacitation sperm (POS). Finally, a semen sample swim-up was acquired: the pellet was added to 1 mL of Sperm Rinse medium (Vitrolife, Gothenburg, Sweden). The tube was positioned at an angle of 45° and kept for 45 min at 37 °C. Next, the upper phase was gently suctioned, and 20 μL of wash was taken from the sperm swim-up (SU) third group (Modified, [35]), and sperm was prepared by density gradient with modified swim-up.

### 4.3. Semen Sample Storage

Semen samples belonging to 35 patients were separated into three samples per patient. One hundred and five samples were separated in aliquots of 20 µL and stored at −80 °C until analysis.

They were classified as subgroups: the first group had fresh sperm (wash) with low motility, the second group had sperm post-capacitation (POS) with medium motility, and the third group had sperm swim-up (SU) with high motility. All sperm samples were stored.

### 4.4. Nanoparticle Synthesis and Characterization of Gold Nanoparticles

Gold nanoparticles (AuNPs) were synthesized following the protocol described by Hermanson et al., 2008 [36] with some modifications. AuNPs were prepared by chemical reduction. AuNPs were synthesized by mixing 250 µL of a 20 mM AuCl3 solution, 250 µL of 1% *w*/*v* sodium citrate, and 500 µL of 18 MΩ deionized water. The final concentration of Au was five mM. Next, the AuNPs were centrifuged at 10,000 rpm for 4 min. Finally, the precipitate was separated from the supernatant, the supernatant was discarded, and the precipitate was resuspended in the same volume of AuNPs; the color change of the solution indicates the formation of the monodisperse colloidal gold particles. For the characterization of the gold nanoparticles, the scan was conducted between the wavelengths of 350–800 nm in a CLARIO Starplus spectrophotometer (BMG-LABTECH, Offenburg, Germany), and the particle size of the AuNPs solution was determined hydrodynamic radius of the colloidal solution by dynamic light scattering (Zetasizer, Malvern Panalytical, Worcestershire, UK) [37,38].

### 4.5. Instrumentation and Semen Sample Raman Spectra Acquisition

The sperm sample aliquots were unfrozen for approximately 30 min at room temperature. Next, each sample was homogenized before acquiring Raman spectra for the surface Raman enhancement spectroscopy (SERS) measurements.

Acquisition of SERS activity (785 L, Wasatch Photonics (Orlando, FL, USA) spectra for SERS was realized in three steps. First, 5 uL of AuNPs were deposited on a gold sheet, followed by 5 uL of semen sample on a droplet of AuNPs and the mixture using the same tip. Next, the Raman spectra were collected for an acquisition time of 1 s and a power laser of 20 mW. The Raman Spectrometer used in the spectra acquisition was a Wasatch Photonics (Morrisville, NC, USA) to 785 nm with 270–2000 cm^−1^ spectrometer range: High NA, f/1.3 optical design for superior sensitivity and SNR. The measurements were taken for three groups; 105 samples were semen samples of 35 patients. Classification models using the calibration data were developed to classify sample fresh semen (wash), post-gradient (POS), and swim-up (SU).

### 4.6. Statistical Analysis: Discriminant Analysis (PLS-DA) Model

Partial least squares-discriminant analysis (PLS-DA) classification models using the calibration [27] data were developed to classify fresh sperm (wash), post-gradient sperm (POS), and swim-up sperm (SU). The spectra were preprocessed by baseline removal, following Standard Normal Variate scaling (SNV); modeling and preprocessing were conducted using the PLS Toolbox version 7.5.2 (Eigenvector Research Inc., Wenatchee, WA, USA) with the MATLAB R2016b version 7 platform (MathWorks, Natick, MA, USA). The performance of the PLS-DA models was evaluated using the parameters of a confusion matrix, such as sensitivity (SEN) and specificity (SPE) of the calibration, validation, and prediction. SEN represents the number of samples predicted to be in a class divided by the number of samples. SPE represents the number of samples predicted not to be in a class divided by the number of samples not in a class [39,40]. Other parameters were the receiver operating characteristic curve [41] and the area under the curve of ROC or sensing probability (AUC). An analysis of VIP was executed to identify the spectral variables contributing significantly to class discrimination. In this case, a variable is relevant when it has a VIP value greater than 1.

## Figures and Tables

**Table 1:** Semen parameters statistical data.

Fresh Sperm				
Parameter	Mean ± SD	Median	Minimum	Maximum
Age	37.4 ± 5.9	38	26	50
Volume (mL)	2.85 ± 1.41	3	1	8
Motile Spermatozoa (1 × 10^6^/mL)	39.1 ± 33.8	26	1.6	124
Total Concentration (1 × 10^6^/mL)	63.1 ± 47.6	47.6	2.9	185
Motility A %	2.1 ± 5.6	0	0	20
Motility B %	54.4 ± 8.6	53	36	73
Motility C %	12.1 ± 6.3	12	2	25
Motility D %	31.3 ± 10.8	31	13	55
Normal morphology %	2.69 ± 1.68	2	0	9
DFI (Morphology %)	2.01 ± 0.19	2.01	1.64	2.47
**Post-capacitation Sperm**				
Volume	0.96 ± 0.14	1	0.8	1.4
Motile Spermatozoa (1 × 10^6^/mL)	34.8 ± 27.5	30	2	120
Total Concentration (1 × 10^6^/mL)	53.1 ± 38.2	43	4.8	150
Motility A %	12 ± 5.9	10	3	25
Motility B %	55 ± 10.2	58	24	68
Motility C %	14.9 ± 9.9	15	0	54
Motility D %	18.2 ± 7.6	18	0	33
Post-Normal morphology %	5.49 ± 3.12	5	1	14
Post-DFI (% Morphology)	1.82 ± 0.17	1.8	1.45	2.39

**Table 2:** Confusion matrix, SEN, SPE, LV and accuracy.

	Model 1 Confusion Table (CV)		#	LV	SEN	SPE	Accuracy
	POS, SU	WASH		4	0.94	0.86	0.91
Predicted as POS, SU	66	5	70				
Predicted as Wash	4	30	35				
	**Model 2** Confusion Table (CV)		#		SEN	SPE	Accuracy
	POS	SU		6	0.71	0.77	0.74
Predicted as POS	25	8	35				
Predicted as SU	10	27	35				

**Table 3:** Main bands corresponding to the sperm Raman spectrum and their assignments.

Assignments of Raman Peaks Sperm Spectra				
Signals	Sperm Peaks (cm^−1^)	VIP Wash vs. POS + SU	VIP POS vs. SU	Assignments
1	408	9	8.5	S–S stretch, symmetric skeletal vibration Lysozyme
2	728	<1	13.5	deoxyadenine
3	1000	2.5	1.3	Phosphate backbone CH_2_/NH_3_ rocking (Protein)
4	1280	1.3	3.5	DNA PO_4_^3−^ asymmetric stretching
5	1319	3.6	5	Guanine, CH_3,_ CH_2_ wagging nucleic acids. (Protein)
6	1455	2.4	4	CH_2,_ CH_3,_ bending mode. Tryptophane
7	1575	2.8	2	DNA/RNA Guanine, adenine

## Data Availability

The original contributions presented in this study are included in the article. Further inquiries can be directed to the corresponding author.

## Abbreviations

The following abbreviations are used in this manuscript:

RS	Raman spectroscopy
UMI	Dire Unexplained male infertility
WHO	World Health Organization
ART	Assisted Reproductive Techniques
DGC	Gradient Centrifugation
SU	Swim-up
IUI	intrauterine insemination
IVF	in-vitro fertilization
PLS-DA	partial least squares discriminant analysis
wash	washing
POS	post-capacitation
AuNPs	Gold nanoparticles
SERS	surface Raman enhancement spectroscopy
SNV	Standard Normal Variate scaling
SEN	sensitivity
SPE	specificity
ROC	operating characteristic curve
AUC	sensing probability
VIP	variable importance in projection
DLS	Dynamic light scattering
